# Addition Silicone Impressions in Fixed Prosthodontics: Clinical Standpoints

**DOI:** 10.7759/cureus.44014

**Published:** 2023-08-23

**Authors:** Pallavi Madanshetty, Satyabodh S Guttal, Roseline Meshramkar, Prabha S Newaskar, Gouri V Anehosur

**Affiliations:** 1 Department of Prosthodontics, Shri Dharmasthala Manjunatheshwara College of Dental Sciences, Dharwad, IND; 2 Department of Prosthodontics, Rural Dental College, Pravara Institute of Medical Sciences, Loni, IND

**Keywords:** gingival retraction, impression techniques, impression materials, elastomers, vinyl polysiloxane, polyvinyl siloxane, addition silicone

## Abstract

Addition silicones have revolutionized the field of fixed prosthodontics because of their dimensional stability, sufficient tear strength and excellent detail reproduction. This review study aims to provide a detailed description of the essential variables to be taken into account during the process of making addition silicone impressions in fixed prosthodontics. These variables include the selection of appropriate tray type, size, and fabrication; the use of tray adhesive; gingival displacement techniques; manipulation of the impression material; the choice of the impression material's viscosity; impression techniques; and the proper insertion, removal, disinfection, and pouring of the cast. Additionally, this review aims to help doctors produce high-quality impressions by empowering them to critically assess the impressions to spot mistakes and motivating them to redo impressions that have serious problems before submitting them to the laboratory.

## Introduction and background

Since their introduction in the mid-1970s, addition silicone, polyvinyl siloxane (PVS), or vinyl polysiloxane (VPS) impression materials have captured the lion's share of the contemporary market [1]. Owing to their user-friendly nature, superb elastic recovery, excellent reproduction of detail, outstanding dimensional stability, sufficient tear strength, ability to yield multiple accurate casts from a single impression, and positive patient acceptance, these materials have revolutionized the field of fixed prosthodontics [2]. Additionally, the availability of a variety of viscosities enables dentists to select the appropriate consistency that suits a variety of clinical scenarios [3].

Despite the widespread use of VPS impression materials, dental laboratories have reported high incidences of clinically detectable errors in fixed prosthesis impressions received from dentists [4]. According to Al-Odinee et al., Rau et al. and Samet et al., over 85% of fixed partial denture impressions provided to dental laboratories exhibited at least one error [4-6]. Given that impression errors can significantly impact the fit of the final prostheses and consequently influence the treatment outcomes, this concern warrants serious attention. It is postulated that the faulty impressions do not arise from inherent deficiencies in the impression materials but rather from dentists' lack of knowledge and skills in handling the material [1]. Therefore, in addition to understanding the physical characteristics of the impression materials, dentists must also meticulously consider various other factors to ensure the production of error-free impressions [7]. The significant factors for a successful impression are discussed next.

## Review

Selection of type, size and fabrication of the tray

Custom Trays vs. Stock Trays

During the setting process, the elastomers undergo polymerization shrinkage, which is proportional to the thickness of the impression material [1]. To minimize the shrinkage and its associated deformation, it is advisable to use a reasonably thin and uniform layer of material, typically around 2 mm in thickness [8]. Stock trays, being generic, may not adapt optimally to the patient's arches, leading to variations in material thickness and potentially resulting in dimensional errors [9]. In contrast, custom-made trays offer a clear clinical advantage as they ensure a consistent and uniform thickness of the impression material, thereby enhancing dimensional accuracy and stability [8-10]. In addition, the closely fitted design of the custom tray reduces patient discomfort during the impression procedure and minimizes the volume of material required for making the impression. Consequently, the use of custom trays not only mitigates polymerization-related shrinkage but also compensates for the additional cost incurred in their fabrication [8]. Therefore, it is recommended to employ custom trays in all fixed partial denture cases, or at the very least, in cases involving multiple preparations (three or more units) requiring impressions of the entire dental arch [11].

Fabrication of Custom Trays

Custom trays are constructed on the diagnostic cast using auto-polymerizing or heat-activated polymethyl methacrylate (PMMA) resin, thermoplastic resins, or light-cure bis-acrylic materials [8]. To achieve a uniform thickness of the impression material, two layers of baseplate wax are applied to the cast [12]. The wax is shaped to include occlusal stops (tooth stops) that aid in the proper orientation of the tray in the patient’s mouth. Preferably, three occlusal stops are placed on the non-functional cusp tips of the teeth, with at least one stop positioned behind the prepared tooth. However, if the occlusal stop is present on the most distal prepared unit, spatial orientation may be lost, and the required material thickness may not be achieved. In such circumstances, a tissue stop can be created before the tooth preparation, either in the patient's mouth or on the cast, using low-fusing impression compound or putty [13]. To facilitate easy removal of the wax spacer from the tray, tin foil is draped over it. Without this precaution, the wax spacer may melt due to heat released during the polymerization of the tray material, leaving behind a residue that may hinder the bonding of the tray adhesive to the tray [9]. Making PMMA trays at least 24 hours in advance allows for complete polymerization and shrinkage processes, guaranteeing their stability [11]. Notably, light-activated trays are reportedly more stable than conventional materials [1].

Despite the strong recommendation for using custom trays in full arch, a survey by Shillingburg et al. revealed that nearly three-fourths of the clinicians utilize stock metal trays in their practice, possibly due to convenience [14]. The advantage of stock trays over custom trays lies in the ability to make impressions in the same visit without the need for primary impressions, study models, or the additional cost of custom trays. However, the disadvantage arises when the principle of bulk control is disregarded, leading to suboptimal impressions. Nonetheless, if dentists opt to use stock trays, the following guidelines may be followed.

Stock tray selection

Selection of Tray Material: Metal Tray vs. Plastic Tray

Plastic impression trays are commonly used due to their affordability, possibly leading to their widespread use, or it could be attributed to clinicians' unawareness of their limitations [6]. However, plastic trays have a higher propensity for flexure, increasing the risk of the impression material separating from the adhesive during polymerization and upon removal from the oral cavity, which can result in significant distortion of the impression [15]. In contrast, metal impression trays offer greater rigidity and accuracy compared to plastic trays. Therefore, when a high level of precision is required, metal trays should always be the preferred choice.

Selection of Tray Size

Careful consideration should be given to selecting a tray that adequately covers all teeth, including the distal most teeth, without contacting the soft tissues while allowing a minimum of 2-3 mm of the material between the tray and tissues [16]. Underextension of the tray may prevent the impression material from capturing the tissues apical to the preparation, thereby hindering the technician from designing an emergence profile for the crown. Gingival overextension that extends more than 2-3 mm beyond the gingival crest may result in excessive permanent deformation of the impression material during removal [8].

Modification of the Stock Tray

For full-arch impressions, posterior damming of the impression tray is essential to prevent flow defects and gag reflexes by restraining the impression material from flowing beyond the tray. If posterior damming is not performed, the impression material may flow distally, resulting in tear-shaped voids on the distal part of the posteriormost teeth [13,17]. In cases where the patient’s palate is high and narrow, the palatal vault of the maxillary tray should be blocked with VPS putty. Otherwise, the impression material can flow into the deep palate, failing to generate sufficient hydraulic pressure to force the material into the gingival sulcus, leading to defects in the form of deep grooves extending from the palatal tooth necks of the posterior teeth to the palate.

Adhesion of the Impression Material to the Tray

Due to the rearrangement of bonds during the polymerization reaction, elastomers tend to shrink towards the center of the mass. Among all elastomers, additional silicone exhibits the least amount of shrinkage, approximately 0.15%, followed by polyether with 0.2% [2]. Without the tray adhesive, polymerization shrinkage would proceed unchecked, resulting in a die that is small in height and diameter [18]. It is preferable to produce a die with a larger diameter by using tray adhesive to direct shrinkage toward the walls of the impression tray. Additionally, during the removal of the tray, especially from the deep undercuts, the impression material may pull away from the tray if it is not well-bonded to it. Hence, it is mandatory to apply the tray adhesives to chemically bond VPS impression materials to the tray.

The adhesives often employed for VPS impression materials contain polydimethylsiloxane and ethyl silicate in a volatile solvent in the form of ethyl acetate [19]. Polydimethylsiloxane chemically bonds with the silicone impression material, whereas ethyl silicate produces hydrated silica that physically bonds to the impression tray [20]. Ethyl acetate reacts with the auto-cured tray material to create micro-irregularities so that the adhesive bonds with it mechanically [16]. The solvent also swells the surface of the tray, making it easier for the adhesive to interact with and penetrate the tray [21]. Before loading the impression material into the tray, the solvent must completely evaporate, leaving a thin film of adhesive. Drying the adhesive in the open air for 10-15 minutes ensures complete evaporation of the solvent [20]. It is best to avoid using compressed air or an air blower to hasten the drying process since doing so might result in insufficient solvent evaporation, and hence, a weak bond. The adhesive should be applied minimally all over the intaglio surface of the tray and extended about 2 mm over the tray borders to ensure that the impression material at the borders stays bonded to the tray [22]. Two thin coats of the adhesive should be painted on the tray, with the first coat being allowed to dry before applying the second. When using paint-on adhesives with a reusable brush, precautions should be taken to prevent cross-contamination [23]. After the tray adhesive has been applied, if the impression tray is placed in the mouth to verify it, saliva contamination occurs, and the bond strength of contaminated adhesives may be decreased by one-fifth. Therefore, to maintain a good bond strength, tray adhesives should be reapplied. It is important to note that, although VPS tray adhesives have a lower bond strength compared to other elastomers, they cannot be used interchangeably [19].

Hydrophilicity of VPS

Ideally, impression materials should exhibit hydrophilic properties. However, VPS materials were originally hydrophobic in nature, leading to changes in pouring bubble-free casts and obtaining full-arch impressions without voids [2,24].

Efforts have been made to improve the hydrophilicity of VPS impression materials by adding external and/or internal surfactants to reduce contact angle and enhance wettability. Although these surfactants reduce the surface tension of the liquid, they do not affect the physical properties of VPS. Accordingly, relatively bubble-free casts can be achieved, but accurate impressions cannot be made in a wet environment [24]. Consequently, despite the claims of increased hydrophilicity, it should be emphasized that accurate impressions can only be obtained in dry conditions [1].

Maintaining a Dry Field

Since the newer VPS materials are not completely hydrophilic, effective moisture control is crucial for a successful fixed partial impression, particularly in critical areas such as the sulcus. Otherwise, voids at the margins, which are the most prevalent error in VPS impressions, become inevitable. If the gingiva is unhealthy, it may bleed profusely following minimal trauma during tooth preparation, making it difficult to maintain a dry field. For this reason, periodontal therapy should be performed in advance to allow the soft tissues to heal before planning a fixed prosthesis. Patients should be advised to use the 0.12% chlorhexidine mouthwash twice a day for two weeks before receiving multiple crowns or fixed prostheses. When the soft tissues are pink and firm, bleeding during tooth preparation will be minor or nonexistent [25]. Furthermore, proper gingival displacement techniques and explicit hemostatic medications should be employed to maintain a dry gingival sulcus.

Gingival Displacement

Adequate gingival deflection is imperative for the subgingival finish line to enable the impression material to register a 360-degree circular profile and capture some tooth structure beyond the finish line to create the emergence profile. Gingival displacement procedures can be categorized as mechanical, chemical, surgical, or combinations of these three techniques [26,27]. The common practice involves the mechanical-chemical displacement technique, which employs gingival retraction cords and certain hemostatic medications [28]. Surgical techniques, such as rotary gingival curettage and electrosurgery, are less commonly used and are typically combined with mechanical-chemical techniques. Two main variations of the mechanical-chemical method for gingival displacement are the single-cord method and the double-cord method [28].

Single-cord technique: The technique is commonly used when one to three teeth with healthy gingiva and margins less than 0.5 mm subgingivally need to be recorded [1,29]. In this method, only one cord of the largest possible diameter that does not cause trauma to the sulcus epithelium is used. The use of a larger cord creates enough gingival displacement to accommodate the bulk of the impression material, resulting in a thick and round-edged sulcus reproduction as opposed to a feather-edged one, which is often observed with thinner cords. The feather-edged wash impression material often tears upon impression removal or distorts during die-pouring procedures and should be avoided [30]. The selected cord is impregnated with a retraction solution, and the excess solution is removed with an absorbent cotton pad before packing the cord atraumatically into the sulcus. Commonly used retraction solutions include epinephrine, aluminum potassium sulfate, aluminum sulfate, and aluminum chloride [28]. However, epinephrine is contraindicated in patients who are on beta-blockers and antihypertensive medications, and those receiving treatment for depression with tricyclic antidepressants and monoamine oxidase inhibitors (MAO) [31]. Epinephrine can increase blood glucose levels, making it also contraindicated in diabetic patients [32]. Owing to its significant negative systemic effects, epinephrine should be used with great caution. There is speculation that VPS and polyether impression materials may be inhibited by astringents and retraction solutions containing adrenalin and ferric sulfate, and therefore, should be avoided [33]. The impregnated cord is kept in the sulcus for 8-10 minutes to allow enough time for necessary hemostasis and gingival displacement. The impression is then made after removing the cord, which must be wetted with water before removal to avoid traumatizing the inner epithelial lining of the sulcus and causing bleeding [28].

Double-cord technique: After the initial supra-gingival tooth preparation, a small diameter cord with no hemostatic agent is inserted in the sulcus [28]. This cord pushes the gingiva apically, preventing trauma to the gingival epithelium caused by the rotary cutting instruments during subgingival preparation. Following the subgingival margin preparation, a second cord of a larger diameter, soaked in the hemostatic agent, is placed in the sulcus above the small-diameter cord [25]. After waiting for 8-10 minutes, the second cord is removed, leaving the smaller cord in situ, and the impression is made. Leaving the small cord in place allows it to absorb moisture from the gingival crevice and prevents tissues from collapsing on the preparation. Ideally, the small cord should stay in the sulcus after the impression has been removed rather than getting incorporated into the impression [34]. If not, the impression material may tear when the impression is removed. Cutting the small cord so that both ends perfectly meet in the sulcus is one method to prevent the cord from being removed with the impression, as cords that are too short or too long will be incorporated into the impression. Another method is to prevent the cords and sulcus from drying out [28,34].

Viscosity selection

VPS impression materials are available in different viscosities, allowing them to be selected to suit various clinical situations. The quantity of inert fillers in the material contributes significantly to the variation in viscosities, with viscosities ranging from putty to heavy-, medium-, or light-body materials [35].

Two general principles for choosing the right viscosity materials are as follows: (a) lower viscosity results in better fine detail reproduction, but (b) the greater the polymerization shrinkage during the setting reaction, the better the impression. Therefore, the best strategy for making a good impression is to select low-viscosity material in the least permissible thickness to record fine details of the tissues, while the bulk of the impression should be made with high-viscosity tray materials to minimize dimensional changes caused by polymerization shrinkage [1].

Light Body (Syringe or Wash Materials)

The viscosity of these products is the lowest since they contain the fewest fillers. As a result, they flow readily and reproduce fine detail down to 25 μm or less, meeting the requirements of the American Dental Association specification [1]. Earlier light-body materials had outstanding flow properties, but after being syringed into the prepared teeth, they tended to drip off, particularly if the tooth was in the upper arch [36]. In order to address this challenge, most of the newer VPS products are now thixotropic. This property allows them to stay in place where they are syringed but flow readily when the heavier body tray materials are placed on top of them [1].

Light body materials cannot be used alone because they exhibit a large amount of polymerization shrinkage and related dimensional changes, attributed to their small filler and large polymeric content. Additionally, their stiffness is not sufficient to resist deformation when poured with die materials [1,37]. Therefore, they are used in combination with putty or heavy-body tray materials.

Medium/Regular Body (One-Step Monophasic Materials)

The medium body is commonly used as a monophase material in single-viscosity or monophase techniques. These materials are pseudoplastic in nature and exhibit shear-thinning properties. This means that under high shear loads, these materials show a reduction in their relative viscosities. As a result, a medium-body impression material may possess just the right amount of viscosity to prevent drip-off or excess flow when loaded into an impression tray. However, it can also exhibit an apparent lowered viscosity suitable for subgingival impressions when expressed through an impression syringe tip [19].

Heavy Body (Tray Material)

Owing to their higher viscosity, heavy-body materials are typically used as tray materials to support light-body materials. They provide bulk and rigidity to the impression and create hydraulic pressure to drive the lower viscosity impression material into the gingival sulcus. Heavy-body materials have been shown to have higher tensile strength, and as a result, better tear resistance than those with a light body consistency [35].

Putty (Very High Viscosity Material)

Putty materials experience the least shrinkage during polymerization because of their high filler content. However, they can only replicate fine details down to 75 μm [1]. As a result, they cannot be utilized to capture the impression on their own; instead, they are used to support light-body materials, which is referred to as putty-wash techniques.

Manipulation of VPS

Currently, elastomeric impression materials are available in three distinct modes of mixing: manual mixing, static mixing, and dynamic mechanical mixing [16]. The advent of auto-mixed, cartridge-based addition polymerizing silicones represents a noteworthy development in contemporary times [36]. Some reasons why auto-mixing is better than manual mixing are as follows: (a) it eliminates the need for spatulation and reduces associated operator errors [38]; (b) the quantity of voids generated is notably reduced by a factor of four to five when contrasted with manual mixing, resulting in more precise impressions; (c) auto-mixing has been found to be more cost-effective, with only one-third of the material being wasted and (d) it reduces the required time and minimizes discomfort and inconvenience for the patient, even when a novice operator is in charge [39].

Setting Reaction

The setting reaction of VPS impression materials involves cross-linking vinyl siloxane in the base material with hydrogen siloxane in the reactor through a chloroplatinic acid catalyst, without any concomitant formation of byproducts [2]. As the reaction proceeds, longer and branched polymer chains are formed, resulting in a three-dimensional network. During this reaction, hydrogen is produced, and then it is removed by palladium and platinum.

Interaction of VPS Impression Materials With Other Materials

As previously mentioned, a trace amount of a platinum catalyst is used in the setting process of VPS materials. Any factor that affects its efficiency can hinder the polymerization reaction [38]. Usually, sulfur or its derivatives, which are often present in natural latex gloves used during manufacturing, can contaminate the chloroplatinic acid catalyst in VPS and impede the polymerization process [2,40]. Additionally, polymerization inhibition is observed when a previously gloved hand comes into contact with the impression material or when it is allowed to set in contact with a latex rubber dam. To prevent sulfur contamination, it is recommended that the clinicians avoid touching the unset impression material, tooth preparations, adjacent gingiva, the interior of the tray, the mixing spatula or mixing pad, the end of a mixing tip, and the retraction cord while donning latex gloves. However, handling putty without gloves can be considered, provided rigorous hand hygiene protocols are adhered to. Instead, polyethylene gloves or vinyl gloves are recommended instead of latex gloves. Certain vinyl gloves may also elicit a similar response owing to the presence of a sulfur-containing stabilizer used during their production [2]. Polymerization inhibition may be indicated by an impression that has a thin coat of unset material or a sticky substance on its surface. Although the inhibition may be minimal, the gypsum cast in contact with an unpolymerized material will be distorted, rendering it unsuitable for use [12]. Additionally, polyether and polysulfide impression materials leave a chemical film on the teeth that inhibits polyvinyl siloxanes. If one chooses to make an impression with polyvinyl siloxane immediately after using either of these materials, it results in incomplete polymerization [1,8].

In the event of inadvertent contamination, pumice can be used to clean the tooth preparations before making the impression. However, cleansing with water alone will not sufficiently eliminate the contaminants, making it impossible to prevent unfavorable interactions [1].

Impression techniques

Putty-Wash Technique

In fixed prosthodontics, the most common technique for making impressions using VPS is the double-mix putty-wash technique, which involves combining two materials with different viscosities. This technique allows impressions to be made in one or two steps [41].

Double-mix, two-step putty-wash/reline technique: In this technique, a preliminary putty impression is made, allowed to set, and subsequently relined with the wash material [37]. There are three main ways of making two-step putty-wash impressions.

The first method involves making an impression with the putty before beginning tooth preparation. The impression is then selectively alleviated to create space for the light-body material before being relined with the wash material. This method exhibits certain drawbacks: (a) undercuts or projections in the putty may cause inaccuracies in tray reseating. Undercuts and projections into interproximal gingival embrasures should be cut away using a no. 15c or no. 11 scalpel blade to permit precise and easy reseating of set putty impressions [42]. (b) Inadequate venting of surplus wash material creates significant hydrostatic pressure at the margin, resulting in a rebound effect when the impression is withdrawn from the mouth after it has set. Therefore, the excess wash material should be vented away by cutting escape ways into the putty from the gingival margin to the tray's outer border with a putty cutting knife [42].

In the second method, a polyethylene sheet is placed between the teeth and the putty material in the tray, preventing the putty from entering interproximal regions and circumventing putty projections into embrasures [12]. Because of the absence of discernible landmarks, guiding planes, or posterior stops within the putty impression, precise reseating of the tray during the wash impression is challenging, and a tray that has been repositioned arbitrarily may not provide a uniformly consistent wash space.

The third method involves making an initial putty impression with resin temporary restorations in situ on the prepared teeth. Upon removal of the impression, the provisional restorations are separated from the set putty, thereby generating a space in the putty corresponding to the dimensions of the provisional restoration [42]. Following that, a wash impression is made.

In general, when employing any of the aforementioned reline methods, it is imperative to ensure that the impression is suitably relieved and possesses sufficient escape ways. Disregarding this precautionary measure may result in the set putty material being compressed if an active force is applied while the wash material is setting. In this situation, the putty rebounds and distorts as soon as the impression is taken out of the mouth. This is the rationale behind holding all full-arch impressions passively when setting [43]. It is important to note that these distortions remain inconspicuous until the castings made from the impression fail to seat [28], and this issue can be somewhat resolved by utilizing a VPS putty material that exhibits inelastic properties upon setting [44].

Double-mix, one-step putty-wash technique (simultaneous technique): Also known as the simultaneous or one-step technique, the putty and light-body materials are mixed at the same time and used in a single step [41]. The wash material is syringed around the prepared teeth, and air is softly blown over it to assist the wash material in reaching the gingival sulcus and to reduce the number of subgingival voids. A burst of air also reduces the number of voids subgingivally [30,45]. The tray loaded with the putty is then placed on top of the unset wash material, so that both materials set simultaneously [37]. Despite the simplicity of this technique’s advantages, there are several associated drawbacks: notably, the high viscosity of the putty has a tendency to displace the unset light-body material from the prepared teeth, and thus, putty rather than the light-body material may record important areas such as the finish line [12,46]. Furthermore, when the putty and syringe materials undergo polymerization concurrently, the polymerization shrinkage of the putty is also accounted for in the total shrinkage of the impression. While the distortion related to polymerization shrinkage is relatively small, it is desirable to avoid it if possible [28]. Additionally, if the putty that has been partially set is placed on the wash material, it may undergo elastic compression. Once removed from the mouth, the impression will “spring back” or relax, and the dies made from this impression will be too narrow and too short [16].

Heavy- And Light-Bodied Material in a Stock or Customized Tray

Except for using a heavy-body material in place of putty, this technique is quite similar to the putty-wash impression technique. The benefits of using a heavy-body material over putty are as follows: (a) a heavy-body material will not move the teeth in the periodontium during the impression process since it has a lower viscosity than putty. This might occur when using particularly viscous putty and lead to erroneous results. (b) The higher viscosity of putty has the potential to flex a tray that lacks rigidity, thereby resulting in impression distortion. (c) Putty frequently pushes the light-bodied material away from the preparation. (d) The capacity of putty to record surface detail is much inferior to that of a light-bodied material, whereas a heavy-body material reproduces surface detail equivalent to a light body [22].

Monophase/Single-Mix, Single-Step Technique

This technique is called a monophase technique because a single viscosity of the impression material is used both as the tray and syringe material. The universal viscosity (medium body) VPS impression material is syringed around the preparation and is also utilized to load the tray. A single viscosity material may not yield the same degree of accuracy as combining low- and high-viscosity materials. However, the detectable accuracy differences are so negligible that they are unlikely to have any clinical significance [28].

Dual-Arch Impression Technique

It allows the dentist to simultaneously make an impression of the prepared teeth and the adjacent teeth and precisely record the inter-occlusal relationship. It is quicker, more comfortable for the patient, and requires less material. The following are the indications for this technique: (a) the presence of a maximum of two prepared teeth, with unprepared teeth located in front and behind them (it is important to note that if the posterior-most tooth in the dental arch is prepared, there may be a reduction in the vertical dimension of occlusion upon closure, resulting in an unfavorable occlusal discrepancy); (b) stable and consistent intercuspal relation and the patient's ability to close into maximum intercuspal position with the tray in place; (c) presence of anterior guidance; (d) no impingement of teeth or soft tissue by the tray; (e) adequate space behind the distal-most tooth in the quadrant to allow for the posterior bar of the tray to fit properly when the mouth is fully closed; if not, the patient can occlude on the bar, resulting in an increased occlusal vertical dimension. It is also very important that the canines be recorded in the impression.

Proper selection of the tray holds significant importance for a dual-arch impression. It is advisable to refrain from utilizing plastic-mesh trays as they possess a high degree of flexibility, which may result in the deformation of the impression. Adequate horizontal clearance between the vertical rim of the tray and the alveolar ridges is necessary.

It has been observed by Bass and Kafalias that achieving maximum intercuspation may not be possible during the dual-arch impression technique owing to the presence of a mesh fabric that separates the teeth [47]. Furthermore, the flexible nature of elastic impression materials and their tendency to regain shape after occlusal pressure is removed may lead to distortion in the impression. Despite criticism, Bass and Kafalias [47] and Pande and Parkhedkar [48] reported favorable results with the use of dual-arch impressions, and this approach is advised for instances involving a small number of units, typically one or two.

It has been observed that no clinical study has demonstrated the superiority of one impression technique over another, as long as the techniques were used appropriately for a given clinical situation. While there may be some disparities in accuracy between the various impression techniques, their magnitudes are not substantial enough to warrant a strong recommendation of one technique over the other [48]. Therefore, it is important to remember that the success of an impression depends more on the skills of the clinician and their level of familiarity with a particular method, than on the selection of a specific technique [49].

Tray placement

Caution should be exercised to position the impression tray centrally. The non-uniform thickness of the impression material in both stock and custom trays is a consequence of the eccentric orientation of the tray to the arch. With custom trays, it is very common to notice excessive thickness of the material on the occlusal surface due to incomplete seating [8], whereas overseating with a minimal occlusal thickness of the material is commonly seen with stock trays.

VPS impression materials exhibit a diminishing flow within the first minute of mixing [50]. Therefore, they allow very little time to adjust the position of the tray in the mouth without generating drags and pulls. Additionally, these materials do not completely cease to flow for another 2-2.5 minutes. Hence, it is of utmost significance to appropriately orient the tray immediately after its insertion into the oral cavity and to hold it stable until it is fully set [51].

Removal of the impression

The set impression material will be compressed in areas of undercutting when it is removed from the mouth. After removal, the impression should ideally be restored to its original dimensions with no permanent deformation. However, no impression material shows 100% elastic recovery. VPS materials offer the best recovery rate of all elastomers at over 99%, with just 1% permanent distortion. It has been observed that the deformation is proportional to the depth of the undercut [1], and therefore, in order to maximize elastic recovery, undercuts must be removed or blocked out before making the impression [33]. Large embrasure spaces should be blocked out using utility wax to prevent distortion of the impression on withdrawal, possible separation of the impression material from the tray, or damage to teeth with compromised periodontal support [42]. If methacrylate-based materials, such as composites, compomers, or resin-modified glass ionomer cements, are used to eliminate the undercuts in the prepared tooth, the impression should not be recorded in the same visit because the impression material will not polymerize adjacent to these restorative materials [36].

It is recommended that the VPS impression be removed until the polymerization has progressed sufficiently to produce an acceptable degree of elasticity. Longer retention in the mouth leads to permanent deformation [7]. VPS materials are highly elastic and have a high strain tolerance, making them easy to remove. In contrast, polyether impression materials are rigid, making removal of undercuts challenging and increasing the risk of die breakage [52].

VPS impression materials are viscoelastic, and the duration of load application affects the deformation. Therefore, to minimize permanent deformation, the VPS impression should be removed as quickly as possible. This involves breaking the peripheral seal between the tissues and the impression in the buccal sulcus by gently pushing the impression with the index fingers of both hands or by blowing air from an air syringe into the buccal sulcus. Removal of the impression parallel to the long axis of the prepared teeth also minimizes the distortion [53].

After removing the impression from the mouth, any material extending beyond the tray should be trimmed back to the extent of the impression tray to prevent distortion caused by the weight of the die material and a lack of tray support [36].

Evaluation of the impression

The impression should be thoroughly evaluated for completeness, wash material thickness, presence of voids or folds, pulls or drags, and homogeneity of the mixed impression material, as well as adherence of the impression material to the tray. A good impression will have a thin, uniform layer of the wash material completely covering the putty. A thick wash material can result from the following: insufficient escape routes, excessive wash material usage, inadequate pressure while seating the tray, or exceeding the working time, causing the wash material to partly polymerize at the moment the tray is inserted.

Voids mean interruption of the surface continuity regardless of its shape, size, or origin and can occur in the impression (a) during mixing of the base and catalyst (usually during manual mixing), (b) during loading of the impression tray or syringe, (c) because of improper syringing technique and (d) because of surpassed working time where there is an impaired material flow [54]. In addition, voids at the margin can be caused by the presence of saliva or blood around the preparation or by insufficient retraction.

To minimize void formation, the mixing tip should be fully immersed in the wash material during syringing with a steady flow rate. Smaller intraoral tips result in noticeably fewer voids [54]. Nonetheless, it is emphasized that the efficiency of the operator holds greater significance in reducing voids as compared to the syringe or the impression material employed [55]. To fill voids on the impression surface, a freshly mixed material should never be added to the previously polymerized material because doing so causes considerable distortion [56].

The impression should be redone if the following errors are observed: (a) obvious streaks of unmixed base or catalyst components in the impression; (b) the presence of voids or folds, particularly in the important area of the impression; (c) if the impression material gets separated from the tray; (d) in the event that the impression tray shows through the impression material in crucial areas. The impression is acceptable in such a case, only if the tray exposure occurs in a narrow spot that is away from the prepared teeth [57].

Disinfection of the VPS impression

Once the impression is removed from the mouth, it should be rinsed under running water to eliminate saliva and blood, which significantly reduces the microbial count. The impression should then be dried and disinfected. The standard disinfection regime of a 10-minute immersion in 0.5% sodium hypochlorite will provide an intermediate level of disinfection and will not have any effect on the dimensional stability of VPS materials. They continue to be stable even when high-level disinfection procedures, such as extended immersion in glutaraldehyde solution for 30 minutes, are employed to manage patients with known cross-infection risks [36]. However, a warning was issued against the use of immersion disinfection for hydrophilic addition silicone impressions due to the possibility of distortion. Instead, the utilization of spray disinfection with a glutaraldehyde solution was recommended, followed by storage in a plastic bag [57].

Pouring of the cast

The dimensional stability of the impression material plays a crucial role when there is a delay in pouring the cast. The newer VPS impression materials have excellent dimensional stability and are least affected by delays in pouring. They are accurate even when poured after one week [14]. After the impression is taken out of the mouth, the newer materials are said to be able to be poured in five minutes. However, it is advised to delay the pouring for at least 30 minutes to allow the setting reaction and visco-elastic recovery to be complete [2].

Hasty pouring of old VPS impression materials produces porosities on the surface of gypsum dies due to the release of hydrogen gas bubbles, thereby necessitating a recommendation to defer pouring for a duration of one hour subsequent to impression making. The purification process, precise proportioning of the base and reactor, as well as the use of palladium as a hydrogen scavenger resolve this issue [19].

While multiple pours of the VPS impression may be deemed acceptable, it is important to note that the precision of second pour casts is consistently inferior to that of the first pour, irrespective of the method employed. It is therefore recommended that the margins of crowns be meticulously finalized on the first die [44].

## Conclusions

In conclusion, accurate and error-free impressions are vital to the success of fixed partial restorations. However, the quality of impressions sent to laboratories is often subpar. This deficit is attributable to a lack of knowledge about the characteristics of the impression material and the necessary skills to handle it, rather than an inadequacy in the impression material itself. As a result, the clinicians are encouraged to adhere to the principles of impression making, familiarize themselves with the impression material and impression techniques, and use them appropriately to maximize the outcome.

## References

[1] Donovan TE, Chee WW (2004). A review of contemporary impression materials and techniques. Dent Clin North Am.

[2] Rubel BS (2007). Impression materials: a comparative review of impression materials most commonly used in restorative dentistry. Dent Clin North Am.

[3] Lu H, Nguyen B, Powers JM (2004). Mechanical properties of 3 hydrophilic addition silicone and polyether elastomeric impression materials. J Prosthet Dent.

[4] Al-Odinee NM, Al-Hamzi M, Al-Shami IZ, Madfa A, Al-Kholani AI, Al-Olofi YM (2020). Evaluation of the quality of fixed prosthesis impressions in private laboratories in a sample from Yemen. BMC Oral Health.

[5] Rau CT, Olafsson VG, Delgado AJ, Ritter AV, Donovan TE (2017). The quality of fixed prosthodontic impressions: an assessment of crown and bridge impressions received at commercial laboratories. J Am Dent Assoc.

[6] Samet N, Shohat M, Livny A, Weiss EI (2005). A clinical evaluation of fixed partial denture impressions. J Prosthet Dent.

[7] Klooster J, Logan GI, Tjan AH (1991). Effects of strain rate on the behavior of elastomeric impression. J Prosthet Dent.

[8] Bomberg TJ, Hatch RA, Hoffman W Jr (1985). Impression material thickness in stock and custom trays. J Prosthet Dent.

[9] Terry DA, Tric O, Blatz M, Burgess JO (2010). The custom impression tray: fabrication and utilization. Dent Today.

[10] Ceyhan JA, Johnson GH, Lepe X (2003). The effect of tray selection, viscosity of impression material, and sequence of pour on the accuracy of dies made from dual-arch impressions. J Prosthet Dent.

[11] Goldfogel M, Harvey WL, Winter D (1985). Dimensional change of acrylic resin tray materials. J Prosthet Dent.

[12] Chee WW, Donovan TE (1992). Polyvinyl siloxane impression materials: a review of properties and techniques. J Prosthet Dent.

[13] Moon MG, Holmes RG (1997). Tissue stops aid in improving accuracy of impressions. J Prosthet Dent.

[14] Shillingburg HT Jr, Hatch RA, Keenan MP, Hemphill MW (1980). Impression materials and techniques used for cast restorations in eight states. J Am Dent Assoc.

[15] Ceyhan JA, Johnson GH, Lepe X, Phillips KM (2003). A clinical study comparing the three-dimensional accuracy of a working die generated from two dual-arch trays and a complete-arch custom tray. J Prosthet Dent.

[16] Anusavice KJ, Shen C, Rawls HR (2013). Phillips’ Science of Dental Materials. Ralph Rawls, PhD. 12th ed. St. Louis: Elsevier.

[17] Haubenreich JE, Osborne PB (2005). Modification of a metal stock tray for a polyvinylsiloxane impression. J Am Dent Assoc.

[18] Ashwini BL, Manjunath S, Mathew KX (2014). The bond strength of different tray adhesives on vinyl polysiloxane to two tray materials: an in vitro study. J Indian Prosthodont Soc.

[19] Mandikos MN (1998). Polyvinyl siloxane impression materials: an update on clinical use. Aust Dent J.

[20] Kothari RN, Arpudaswamy S, Nandini Y, Dhole RI, Shivram D, Shetty R (2019). Effect of time and method of drying on bond strength of tray adhesives with vinyl polysiloxanes. J Contemp Dent Pract.

[21] Ona M, Takahashi H, Sato M, Igarashi Y, Wakabayashi N (2010). Effect of reactive adhesives on the tensile bond strength of polyvinyl siloxane impression materials to methyl methacrylate tray material. Dent Mater J.

[22] Bonsor SJ, Pearson GJ (2013). A Clinical Guide to Applied Dental Materials. https://booksite.elsevier.com/9780702031588/about_book.php.

[23] Paczkowski I, Stingu CS, Hahnel S, Rauch A, Schierz O (2021). Cross-contamination risk of dental tray adhesives: an in vitro study. Materials (Basel).

[24] Rameez M, Razi S, Farhan F, Kumar B, Rashid H (2018). Clinical implications of elastomeric impression materials used for complete denture construction. Dent Med Res.

[25] Christensen GJ (2007). Laboratories want better impressions. J Am Dent Assoc.

[26] Gilboe DB (1980). Mechano-chemical gingival displacement. A review of the literature. J Can Dent Assoc.

[27] Nemetz EH, Seibly W (1990). The use of chemical agents in gingival retraction. Gen Dent.

[28] Donovan TE, Gandara BK, Nemetz H (1985). Review and survey of medicaments used with gingival retraction cords. J Prosthet Dent.

[29] Nemetz H, Donovan T, Landesman H (1984). Exposing the gingival margin: a systematic approach for the control of hemorrhage. J Prosthet Dent.

[30] Going RE (1968). Accurate rubber-base impressions. J Prosthet Dent.

[31] Hilley MD, Milam SB, Giescke AH Jr, Giovannitti JA (1984). Fatality associated with the combined use of halothane and gingival retraction cord. Anesthesiology.

[32] Kumbuloglu O, User A, Toksavul S, Boyacioglu H (2007). Clinical evaluation of different gingival retraction cords. Quintessence Int.

[33] Hamalian TA, Nasr E, Chidiac JJ (2011). Impression materials in fixed prosthodontics: influence of choice on clinical procedure. J Prosthodont.

[34] Baba NZ, Goodacre CJ, Jekki R, Won J (2014). Gingival displacement for impression making in fixed prosthodontics: contemporary principles, materials, and techniques. Dent Clin North Am.

[35] Carlo HL, Fonseca RB, Soares CJ, Correr AB, Correr-Sobrinho L, Sinhoreti MA (2010). Inorganic particle analysis of dental impression elastomers. Braz Dent J.

[36] McCabe JF, Walls AG (2008). Applied Dental Materials, 9th Edition. Iowa: Blackwell Publishing.

[37] Ho W, Lin Seow L, Musawi A (2018). Viscosity effects of polyvinyl siloxane impression materials on the accuracy of the stone die produced. J Clin Transl Res.

[38] Craig RG (1985). Evaluation of an automatic mixing system for an addition silicone impression material. J Am Dent Assoc.

[39] Daou EE (2010). The elastomers for complete denture impression: a review of the literature. Saudi Dent J.

[40] Cook WD, Thomasz F (1986). Rubber gloves and addition silicone materials. Current note no. 64. Aust Dent J.

[41] Merchant A, Maiti S, Ashok V, Ganapathy DM (2020). Comparative analysis of different impression techniques in relation to single tooth impression. Bioinformation.

[42] Marshak B, Assif D, Pilo R (1990). A controlled putty-wash impression technique. J Prosthet Dent.

[43] Cowie RR (2007). Understanding impression materials and techniques. Dent Today.

[44] Bell JW, von Fraunhofer JA (1975). The handling of elastomeric impression materials: a review. J Dent.

[45] Harrop TJ, Middaugh DG (1967). Forced air impression technique. J Can Dent Assoc (Tor).

[46] Chugh A, Arora A, Singh VP (2012). Accuracy of different putty-wash impression techniques with various spacer thickness. Int J Clin Pediatr Dent.

[47] Bass EV, Kafalias MC (1992). Dual-arch impressions. Aust Dent J.

[48] Pande NA, Parkhedkar RD (2013). An evaluation of dimensional accuracy of one-step and two-step impression technique using addition silicone impression material: an in vitro study. J Indian Prosthodont Soc.

[49] Perakis N, Belser UC, Magne P (2004). Final impressions: a review of material properties and description of a current technique. Int J Periodontics Restorative Dent.

[50] Lawson NC, Cakir D, Ramp L, Burgess JO (2011). Flow profile of regular and fast-setting elastomeric impression materials using a shark fin testing device. J Esthet Restor Dent.

[51] Sulaiman TA, Nathaniel C (2022). Diagnosing a failed impression: common errors and how to overcome them. https://www.aegisdentalnetwork.com/cced/2019/03/diagnosing-a-failed-impression-common-errors-and-how-to-overcome-them.

[52] Chai J, Takahashi Y, Lautenschlager EP (1998). Clinically relevant mechanical properties of elastomeric impression materials. Int J Prosthodont.

[53] Nandini VV, Venkatesh KV, Nair KC (2008). Alginate impressions: a practical perspective. J Conserv Dent.

[54] Stackhouse JA Jr, Harris WT, Mansour RM, Von Hagen S (1987). A study of bubbles in a rubber elastomer manipulated under clinical conditions. J Prosthet Dent.

[55] Kishimoto M, Shillingburg HT Jr, Duncanson MG Jr (1980). A comparison of six impression syringes. J Prosthet Dent.

[56] Bailey LR (1957). Rubber base impression techniques. Dent Clin North Am.

[57] Rosenstiel S, Land MF, Fujimoto J (2016). Contemporary Fixed Prosthodontics, 5th Edition. St. Louis: Elsevier.

